# Molecular Diagnosis of Pneumonia Using Multiplex Real-Time PCR Assay RespiFinder® SMART 22 FAST in a Group of Moroccan Infants

**DOI:** 10.1155/2020/6212643

**Published:** 2020-02-18

**Authors:** Kenza Hattoufi, Houssain Tligui, Majdouline Obtel, Sobha El Ftouh, Aicha Kharbach, Amina Barkat

**Affiliations:** ^1^National Reference Center in Neonatology and Nutrition, Children's Hospital, University Hospital Centre IBN SINA, Rabat, Morocco; ^2^Research Team on Health and Nutrition of Mother and Child, Faculty of Medicine and Pharmacy, Mohammed V University, Rabat, Morocco; ^3^Medical Research Laboratory, Children's Hospital, University Hospital Centre Ibn Sina, Rabat, Morocco; ^4^Department of Public Health, Laboratory of Biostatistics, Clinical Research and Epidemiology, Faculty of Medicine and Pharmacy, Mohammed V University, Rabat, Morocco; ^5^Gynaecology-Obstetrics and Endocrinology Department, Maternity Souissi, University Hospital Center IBN SINA, University Mohammed V, Rabat, Morocco

## Abstract

**Background:**

In Morocco, pediatric pneumonia remains a serious public health problem, as it constitutes the first cause of mortality due to infectious diseases. The etiological diagnosis of acute respiratory tract infections is difficult. Therefore, it is necessary to use Multiplex real-time polymerase chain reaction assay tests in a routine setting for exact and fast identification.

**Objectives:**

In this paper, we present the clinical results of pediatric pneumonia and describe their etiology by using molecular diagnosis. *Study design*: Tracheal secretion was collected from infants presenting respiratory distress isolated or associated with systemic signs, attending the unit of Neonatology between December 1, 2016, and Mai 31, 2018. Samples were tested with the multiplex RespiFinder® SMART 22 FAST which potentially detects 18 viruses and 4 bacteria.

**Results:**

Of the 86 infants considered in this study (mean age 31 ± 19 days) suspected of acute respiratory tract infections, 71 (83%) were positive for one or multiple viruses or/and bacteria. The majority of acute respiratory tract infections had a viral origin (95%): respiratory syncytial viruses (A and B) (49%), rhinovirus (21%), coronaviruses 229E (11%), humain metapneumovirus (5%), influenza A (3%), influenza H1N1 (1%), adenovirus (2%), and parainfluenza virus type 4 (2%). Among our patients, 6% had *Mycoplasma pneumoniae*. Coinfections were not associated with severe respiratory symptoms.

**Conclusion:**

The clinical spectrum of respiratory infections is complex and often nonspecific. Thus, the early and fast detection of related causative agents is crucial. The use of multiplex real time polymerase chain reaction may help choose an accurate treatment, reduce the overall use of unnecessary antibiotics, preserve intestinal flora, and decrease nosocomial infection by reducing the length of hospitalization.

## 1. Introduction

According to the World Health Organization (WHO), pneumonia is defined as acute respiratory tract infections (RTIs) which affect the lungs tissue (bronchi, bronchioles, and alveolar tissue) [1]. In Morocco, pediatric pneumonia remains a serious public health problem and constitutes the first cause of mortality due to infectious diseases [2]. Pneumonia is a major cause of childhood morbidity and mortality worldwide. The WHO estimates that the 1.4 million children who die annually are under 5 years of age, where the greatest risk of death is in the neonatal period [3].

The epidemiology and etiology of pneumonia vary from country to country and from region to region. In developing countries, the incidence of pneumonia among children under 5 years of age is 0.29 episodes per child/year, compared to 0.05 episodes per child/year in developed countries [4, 5].

Pneumonia is caused by a variety of microorganisms (viruses, bacteria, or fungi). Viruses are the most common causative agents for children under 5 years of age, especially respiratory syncytial virus (RSV), rhinovirus (RV), influenza (IV), parainfluenza viruses (PIV), and adenovirus (ADV) [6, 7]. At least 26 viruses have now been associated with pneumonia. Their distribution varies by season, geographic region, and age group (4). Considering the frequency of viral infection, antibacterial therapy is often employed inadequately and unnecessarily [8].

The etiological diagnosis of pneumonia is difficult, due to the similarity in clinical presentation and also due to the overlap of the symptoms between viruses and bacteria, or between different viruses [9]. For this reason, the use of Multiplex real-time polymerase chain reaction (PCR) assays tests in a routine setting for exact and fast identification appears to be necessary. They detect a wide range of viral and bacterial pathogens simultaneously in a single reaction, with higher sensitivity and specificity in hours [10].

The aim of this study is to present the clinical results of pediatric pneumonia and describing their epidemiology and etiology among infants who live in Morocco and admitted to a neonatology unit.

## 2. Materials and Methods

### 2.1. Design and Population of the Study

This prospective study was conducted from December 1, 2016, to May 31, 2018, at the National Reference Center for Neonatology and Nutrition and Medical Research Laboratory at Children's Hospital of Rabat. We included in this study 86 infants admitted with respiratory distress isolated or associated with systemic signs.

### 2.2. Inclusion Criteria

Infants, regardless of their gestational age or geographical origin, whose actual age was less than 4 months, hospitalized for suspicion of pneumonia.

Inclusion criteria were based on diagnosis by clinical symptoms (respiratory symptoms). The infants were recruited if they presented with symptoms associated with the WHO's definition of clinical pneumonia. They are defined as having a history of cough, raised respiratory rate, or chest wall indrawing [2, 11].

### 2.3. Exclusion Criteria

All newborns aged between 0 and 9 days and infants with transient respiratory distress or respiratory distress of cardiac, neurological, or malformative origin, were excluded from this study.

### 2.4. Data Collection

All data were entered into sheet containing epidemiological, clinical, paraclinical data, and clinical evaluation of patient, as well as information regarding the patient's family members.

The studied data are as follows:

- Epidemiological data: age, sex, and socioeconomic status
- Environment of the patient, home conditions, and health history
- Clinical data: symptoms and evolution
- Paraclinical data: biological analysis (complete blood count, C-reactive protein (CRP), RT-PCR), chest X-rays (interpreted by pediatricians)

### 2.5. Collected Samples

The tracheal secretion (TS) samples were collected in the first 12 hours of hospitalization and transferred from the neonatology department to the medical research laboratory at Children's Hospital in Rabat.

The collection of TS was performed according to the standard procedure, that is, by using a tracheal aspiration probe. This was introduced until resistance was encountered and retracted by approximately 2 cm. This was followed by the release of the vacuum, and the probe was delicately removed using turning movements, from which the secretion was aspirated into a sterile collector tube.

### 2.6. Laboratory Methods

The samples were analyzed according to the manufacturer's instructions by using the RespiFinder® SMART 22 FAST. This assay can detect and differentiate simultaneously 22 respiratory pathogens: 18 viruses (influenza viruses A and B, influenza virus A H1N1, respiratory syncytial viruses (RSV) A and B, parainfluenza viruses 1, 2, 3 and 4, rhinovirus (RV)/enterovirus, coronaviruses (Cor) (229E, NL63, OC43, and HKU1), human metapneumovirus (hMPV)), adenovirus (ADV), bocavirus (BoV), and 4 bacteria (*Legionella pneumophila*, *Bordetella pertussis*, *Mycoplasma pneumoniae*, and *Chlamydophila pneumoniae*) [12].

175 *μ*l of TS samples were eluted in 40 *μ*l of elution buffer. The assays comprised a preamplification step, which combines reverse transcriptase and multiplex target amplification PCR, followed by a probe hybridization step, a probe ligation step, and a probe amplification step. The Internal Amplification Control (IAC) was added at the beginning of the procedure to differentiate between samples with true-negative results and samples with false- negative results due to PCR failure. Targets are detected using capillary electrophoresis [13].

### 2.7. Statistical Analysis

Statistical analysis was performed with the Package for the Social Sciences (SPSS.18) and analyzed by using the software RChi2 of Pearson. A *p* value <0.05 was considered statistically significant.

### 2.8. Ethical Consideration

The parents of all participants have given informed written consent. The protocol of the study was approved by the ethics committee of the biomedical research of the Faculty of Medicine and Pharmacy in Rabat, Morocco.

## 3. Results

The subjects of this study were a total of the 86 infants. They presented with respiratory symptoms and suspicion of pneumonia. Among them, 46 (53%) were male and 40 (47%) were female, and the sex ratio was 1.15. The mean age was 31 ± 19 days. 54% of the patients were of middle socioeconomic status, 45% had low socioeconomic status, while only one of them was of a high socioeconomic status. According to the parents, 4 (5%) families had a pet at home, 44 (51%) had dampness at home, and 11 (13%) had lived with smokers (Table 1).

All patients included in this study were conscious and had at least one respiratory symptom with a high frequency of dyspnea, representing 95% of the cases. Cough, rhinorrhea, and fever were frequent with 67%, 62%, and 38%, respectively (Table 2). 84% of our patients performed chest X-ray , where 62% were abnormal. 91% of the cases with abnormal chest X-rays results showed a confirmed infection (*p* < 0.004) using the RT-PCR technique (Table 3). Mean duration of hospitalization was 6 ± 5 days; the length of stay of the dead infants was not included, and it was 10 ± 4 days. During this period, 3% of deaths and 10% of complications by nosocomial infection were detected.

The results of multiplex RT-PCR showed the presence of 7 types of viruses and one species of bacteria. At least, one pathogen was detected in 71/86 of the samples (83%). The most prevalent virus was RSV, detected in 46/86 of the samples (51%), where type A and B represented 59% and 41%, respectively. In the total of the samples, IV A represented 4% whose one patients had a subtype of IV H1N1, RV was detected in 20/86 (23%) of samples, ADV was detected in 2/86 (2%) of samples, Cor229E in 10/86 (12%) of samples, hMPV was detected in 5/86 (6%) samples, and PIV4 was detected in 2/86 (2%) of samples. 6% of the patients had *Mycoplasma pneumoniae*. Coinfection was seen in 22 specimens representing 26% of all samples (22/86) and 31% of positive samples (22/71). In the case of co-infection with two viruses, 19 samples were positive. Coinfection virus/bacteria were detected in 4 cases. The most frequently detected coinfection combinations were RSV/rhino (*n* = 7) and RSV/Cor229E (*n* = 6) (Table 4).

**Figure 1:**
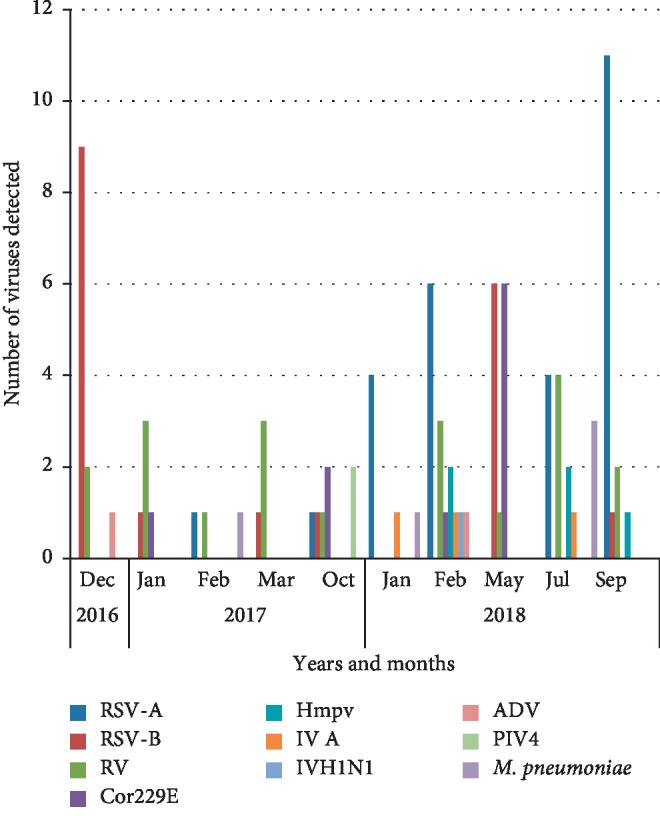
Detection of viruses and bacteria during the study period (2016–2018).

According to our study, the seasonality distribution of viruses varies, the graphic shows that viral infections are essentially present in the coldest season (autumn and winter), when the seasonality of some respiratory viruses is not well established. RSV A and B were the most prevalent viruses; they appeared frequently in September and December. The cases with IV A were detected in January, February, and July; influenza H1N1 was detected in February. Cor229E was most predominant in May. RV was detected during the study without any pick. The other viruses hMPV, ADV, and PIV4 were less frequent (Figure 1).

We compared the epidemiological, clinical, and radiological characteristics between the positive (83%) and negative samples (17%). The proportion of positives in males was 62% (44/71, *p* < 0.001). The rate of prematurity presented 64.3% in the positives cases (*p* < 0.04). For the rest of the general characteristics (including age, socioeconomic status, and environment of the patient), the comparison between the two groups does not show any significance (Table 1). Most clinical signs and symptoms were present in both groups, with a higher frequency in the positive group. The difference was not statistically significant. Nasal flaring was present only in positive cases with a *p* < 0.006 (Table 2).

## 4. Discussion

The human respiratory tract is exposed to viral and bacterial infections. Pneumonia is the major clinical presentation of lower acute respiratory tract infection, which is considered as the major cause of hospitalization among children worldwide. The mortality and morbidity rate is higher especially during the first years of life [14–16].

Several laboratory techniques are used to detect agents responsible for RTIs, including immunofluorescence, enzyme immunoassays, isolation in culture, and molecular biology. The current molecular methods can detect a large panel of viruses, and bacteria also can make the etiological diagnosis more efficient, in contrast to traditional methods which are less sensitive and most of which are not suitable for rapid diagnosis [17–19]. At Children's Hospital in Rabat CHR, RTIs are frequent, especially the acute lower RTIs; they represented a large proportion of all-cause consultations [2]. Therefore, their precise diagnosis is necessary [19]. Currently, a large variety of molecular assays was developed and commercialized [20]. Ruuskanen et al. reported that the evidence of viral infection is present in 49% of pediatric cases using molecular diagnostics [4, 21].

We studied for the first time, prospective data regarding etiology of acute respiratory tract infection in service of neonatology CHR. We evaluated the performance of RespiFinder® SMART 22 FAST in 86 Moroccan infants with clinically suspected pneumonia. A very high percentage of our samples were positive (83%). The majority of our patients had viral infections. Additionally, most viruses in the test panel were detected (RSV, RV, Cor 229E, hMPV, IVA, IV A H1N1, ADV, and PIV4). Dabisch-Ruthe et al. reported that there is a higher clinical sensitivity of the RespiFinder-19 in the detection of virus. They found Respiratory viruses in 64% of the Tracheal secretion (TS) samples [12]. This high positivity is similar to other studies in which molecular methods were performed [2, 22–24].

In this study, the most prevalent pathogen was RSV A/B. It was detected in 53% of samples. According to several studies, RSV (A and B) is known as the most common cause of pneumonia in infants and young children worldwide [25]. Hall CB et al. suggest that RSV has the greatest disease burden both in hospitalized children and in outpatients, especially in children under 5 years of age [26]. This higher proportion in infants is due to maternal antibodies that are ineffective in preventing RSV infections [24]. A systematic review realized by Shi et al. in 2015 estimated that 30 million episodes of acute lower respiratory infection caused by RSV were about a third in the first year of the life among children aged less than 4 years in low-income and middle-income countries. They also estimated 2.8 million episodes in high-income countries [27]. The second virus detected in the study was RV with 23%. The human RV is the most frequent causative agents of both upper and lower respiratory tract infections and the main causative agents of the common cold. It causes severe ARIs in infants and young children [28, 29]. ADV was detected in 2% of cases; it is one of the major causes of lower respiratory tract infection in children aged more than 2 years and in adults, but rarely in newborns and infants [30]. Influenza A accounted for 4% of virus-detection-positive samples. According to the results of a study published in Morocco by Ezzine et al. during both seasons (2016/2017-2017/2018), influenza infections in ARIs cases affects especially adults aged more than 65 years (20.7%) and children aged between 5 and 15 years (36%), while children aged less than two years had a low rate (4.4%) [31]. The IV is known for its seasonal outbreaks of ARI during a few weeks in winter [29]. According to a study realized in Brazil by Canela et al., among children aged between 0 and 18 years, the virus positivity is higher with frequency of IV H1N1 (38%) [32]. PIV subtype 4 was detected in 2% of our cases, and it has been reported to be a much less frequent cause of ARIs [33]. The group of coronavirus is heterogeneous (HCoV 229E, OC43, NL63, and HKU1) [34]. In our study, we detected only Cor229E. The clinical symptoms of coronavirus and other newly discovered viruses involved in RTIs (human bocavirus and hMPV) are similar to the other respiratory viruses and are recognized frequently at young age [13]. Our results are similar to those of other studies conducted among children and infants [35, 36]. Our etiological results coincide with those of a study realized at Toulouze University Hospital among children suffering from ARIs; there samples were analyzed using the RespiFinder 15 assay, the prevalence of virus was 88.7% and the RSV was the most frequently detected 39.5% [9]. Regarding bacteria, only 6% of our patients had *Mycoplasma pneumoniae*, while 81% of the cases have been treated with antibiotic. The low prevalence of bacteria may be explained by the use of antibiotics as an urgent act used in cases suspected with ARTs. Beckmann and Hirsch detected *Mycoplasma pneumoniae* in 2.1% among pediatric and adult patients [36].

According to several studies, the incidence of coinfections depends on different populations and test panels [37]. Bruijnesteijn van Coppenraet et al. reported that the molecular assays revealed a high frequency of double infections. Specifically, coronaviruses (75%), RSV (58%), and RV (46%) were frequently detected as mixed infection [8]. The current study shows coinfection in 31% of positive samples, with a high frequency in the combinations between RSV/rhino and RSV/Cor229E. Several studies had pointed out that clinical severity is associated with coinfection with more than one virus [8, 37, 38]. Another study reported that coinfection was not associated with an increase in the clinical severity [39]. Our results did not show a significant difference in clinical severity between coinfection and single infection. Also, clinical presentation of different causative agents was often similar. The association of viral detection with clinical characteristics shows a predominance of respiratory signs specially (dyspnea, polypnea, cyanosis, labored breathing, wheezing, and Rhonch). Our clinical symptoms and signs concurred with those of the study realized by Singh S et al. among infants aged less than 2 months [40].

## 5. Conclusion

The clinical spectrum of respiratory infections is complex and often nonspecific. Therefore early and rapid detection of related causative agents is crucial. Multiplex RT-PCR assays for respiratory infections are not used in routine diagnosis in Morocco. Their use in the near future may help pediatricians choose an appropriate treatment, reduce the overall use of unnecessary antibiotics, stop their use in patients infected with virus, initiate antiviral therapy and preserve intestinal flora, and help to decrease nosocomial infection by reducing the length of hospitalization, which would also decrease management costs. Multiplex RT-PCR assays for respiratory infections are likely to be the subject of future studies on larger samples and different age groups.

## Figures and Tables

**Table 1:** General characteristics of the recruited patients (December 1, 2016, to May 31, 2018; *N* = 86).

Patient's variables	*n* (%)	Positives cases *n* (%)	Negatives Cases *n* (%)	*p*
Gender				
M	46 (53)	44 (95.7)	2 (4.3)	*0.001*
F	40 (47)	27 (67.5)	13 (32.5)	

Age in hospitalization				
10–30 days	60 (70)	50 (83)	10 (17)	0.4
>30 days–<4 months	26 (30)	21 (80)	5 (20)	

Socioeconomic status				
Low	39 (45)	29 (74.4)	10 (25.6)	0.1
Middle	46 (54)	41 (98.1)	5 (10.9)	
High	1 (1)	1 (100)	0	

Family history				
Asthma	3 (3)	3 (100)	0	0.5
Eczema	2 (2)	2 (100)	0	0.6

Health history				
Prematurity	14 (16)	9 (64.3)	5 (35.7)	*0.04*
Gastroesophageal reflux	1 (1)	1 (100)	0	0.8
Intensive care/oxygen therapy	9 (10)	4 (44.4)	5 (55.6)	*0.007*
Bronchopulmonary dysplasia	3 (3)	3 (100)	0	0.5
Congenital heart disease	1 (1)	1 (100)	0	0.8

Environment of the patient				
Smokers at home	11 (13)	11 (100)	0	0.1
Pets at home	4 (5)	3 (75)	1 (25)	0.5

Home conditions				
Airy and sunny habitat	42 (49)	36 (85.7)	6 (14.3)	0.3
Humidity	44 (51)	35 (79.5)	9 (20.5)	

**Table 2:** Clinical characteristics of hospitalized patients (December 1, 2016, to May 31, 2018; *N* = 86).

	*n* (%)	Positives cases *n* (%)	Negatives cases *n* (%)	*p*
Functional signs				
Dyspnea	82 (95)	67 (81.7)	15 (18.3)	0.4
Cough	58 (67)	47 (81)	11 (19)	0.4
Rhinorrhea	53 (62)	42 (79.2)	11 (10.8)	0.2
Fever	33 (38)	29 (87.9)	4 (12.1)	0.2
Refusal to breastfeed	26 (30)	22 (84.6)	4 (15.4)	0.5
Vomiting	8 (9)	8 (100)	0 (0)	0.2
Diarrhea	2 (2)	2 (100)	0 (0)	0.6
Convulsion	1 (1)	0 (0)	1 (100)	0.1

Physical signs Tone:				
Normal	61 (71)	52 (85.2)	9 (14.8)	0.2
Hypotonic	25 (29)	19 (76)	6 (24)	
Polypnea	56 (65)	45 (80.4)	11 (19.6)	0.3
Tachycardia	28 (33)	22 (78.6)	6 (21.4)	0.3
Bradycardia	3 (3)	1 (33.3)	2 (66.7)	0.07
MAP: normal	86 (100)			
SaO2:				
Normal	81 (94)	68 (84)	13 (16)	0.2
Desaturation	5 (6)	3 (60)	2 (40)	
CRT:				
<3	85 (99)	71 (83)	14 (17)	0.1
>3	0	0	1 (100)	
Pallor	6 (7)	5 (83.3)	1 (16.7)	0.7
Cyanosis	16 (19)	13 (81.2)	3 (18.8)	0.5
Jaundice	6 (7)	3 (50)	3 (50)	0.06
Labored breathing				
Intercostal recession	38 (60)	30 (78.9)	8 (21.1)	0.4
Supraclavicular recession	34 (54)	30 (88.2)	4 (11.8)	0.1
Suprasternal recession	25 (40)	21 (84)	4 (16)	0.4
Nasal flaring	20 (32)	20 (100)	0	*0.006*
Rales				
Wheezing	28 (32)	24 (85.7)	4 (14.3)	0.4
Rhonchi	33 (38)	25 (75.8)	8 (24.4)	0.1
Crackles	3 (3)	2 (66.7)	1 (33.3)	0.4

Cardiovascular signs				
Heart murmur	3 (3)	3 (100)	0	0.5

Abdominal signs				
Hepatomegaly	1 (3)	1 (100)	0	0.8

MAP: mean arterial pressure, CRT: capillary refill time.

**Table 3:** The chest X-ray results (December 1, 2016, to May 31, 2018; *N* = 74).

	*n* (%)	Positives cases *n* (%)	Negatives cases *n* (%)	*p*
Chest X-ray, abnormal	46 (62)	42 (91.3)	4 (8.7)	*0.005*
Primary lung focus	33 (45)	29 (87.9)	4 (12.1)	0.2
Thoracic hyperinflation	15 (20)	15 (100)	0	*0.04*
Bronchiectasis	10 (14)	10 (100)	0	0.1
Horizontalisation of ribs	5 (7)	5 (100)	0	0.3
Cardiomegaly	2 (3)	2 (100)	0	0.6

**Table 4:** Biological results of hospitalized patients (December 1, 2016, to May 31, 2018).

	*n*	%
Anemia (*N* = 70)	19	27

Leucocytes (*N* = 70)		
Leukocytosis	5	7
Leukopenia	23	33

Platelets (*N* = 70)		
Thrombocytosis	15	21
Thrombocytopenia	10	14

C-reactive protein (CRP)		
High CRP (>20 mg/dL)	42	49

Positive viral detection (*N* = 86)	71	83
RSV-A	27	31
RSV-B	19	22
RV	20	23
Cor229E	10	12
hMPV	5	6
IV A	3	3
IV H1N1	1	1
ADV	2	2
PIV 4	2	2

Positive bacterial detection (*N* = 86)		
*M. pneumoniae*	5	6

Single viral detection *(N* = 71)	48	68

Single bacterial detection (*N* = 71)	1	1

Coinfection (*N* = 71)	22	27
RSV-A-RV	3	4
RSV-A-Cor 229E	1	1
RSV-A-hMPV	1	1
RSV-A-IV A	1	1
RSV-A-hMPV-*M. pneumoniae*	1	1
RSV-B-RV	4	6
RSV-B-Cor 229E	5	7
RV-Cor 229E	1	1
RV-hMPV	1	1
RV-*M. pneumoniae*	2	3
hMPV-ADV	1	1
IV A-*M. pneumoniae*	1	1

RSV: respiratory syncytial virus, RV: rhinovirus, Cor 229E: coronaviruses 229E, hMPV: human metapneumovirus, IVA:influenza  A, IV H1N1 : influenza H1N1, ADV: adenovirus, PIV 4: parainfluenza viruse type 4, *M. pneumoniae: Mycoplasma pneumoniae*.

## Data Availability

The data used to support the findings of this study are included within the article.
